# Genome instability-related long non-coding RNA in clear renal cell carcinoma determined using computational biology

**DOI:** 10.1186/s12885-021-08356-9

**Published:** 2021-06-24

**Authors:** Yutao Wang, Kexin Yan, Linhui Wang, Jianbin Bi

**Affiliations:** 1 Department of Urology, China Medical University, The First Hospital of China Medical University, Shenyang, Liaoning China; 2 Department of Dermatology, China Medical University, The First Hospital of China Medical University, Shenyang, Liaoning China

**Keywords:** Genome instability, Long non-coding RNA, Computational biology, Gene set variation analysis, Risk signature

## Abstract

**Background:**

There is evidence that long non-coding RNA (lncRNA) is related to genetic stability. However, the complex biological functions of these lncRNAs are unclear.

**Method:**

TCGA - KIRC lncRNAs expression matrix and somatic mutation information data were obtained from TCGA database. “GSVA” package was applied to evaluate the genomic related pathway in each samples. GO and KEGG analysis were performed to show the biological function of lncRNAs-mRNAs. “Survival” package was applied to determine the prognostic significance of lncRNAs. Multivariate Cox proportional hazard regression analysis was applied to conduct lncRNA prognosis model.

**Results:**

In the present study, we applied computational biology to identify genome-related long noncoding RNA and identified 26 novel genomic instability-associated lncRNAs in clear cell renal cell carcinoma. We identified a genome instability-derived six lncRNA-based gene signature that significantly divided clear renal cell samples into high- and low-risk groups. We validated it in test cohorts. To further elucidate the role of the six lncRNAs in the model’s genome stability, we performed a gene set variation analysis (GSVA) on the matrix. We performed Pearson correlation analysis between the GSVA scores of genomic stability-related pathways and lncRNA. It was determined that LINC00460 and LINC01234 could be used as critical factors in this study. They may influence the genome stability of clear cell carcinoma by participating in mediating critical targets in the base excision repair pathway, the DNA replication pathway, homologous recombination, mismatch repair pathway, and the P53 signaling pathway.

**Conclusion subsections:**

These data suggest that LINC00460 and LINC01234 are crucial for the stability of the clear cell renal cell carcinoma genome.

**Supplementary Information:**

The online version contains supplementary material available at 10.1186/s12885-021-08356-9.

## Introduction

Clear cell renal cell carcinoma (ccRCC) is the most common subtype of renal cell carcinoma, and ccRCC accounts for 80 to 90% of all renal cell carcinomas. ccRCC is a potentially invasive tumor with an overall progression-free survival rate of 70% and a cancer-specific mortality rate of 24% [1]. It is 1.5–2.0 times more common in men than in women. Advanced RCC has a five-year survival rate of 11.7% [2]. Risk factors include smoking, obesity, high blood pressure, chronic kidney disease, and exposure to certain chemicals and heavy metals [3]. The diagnosis of ccRCC has been increasing over the past few years. Although surgery is the most common treatment option, early diagnosis is difficult, and many patients have metastatic disease by this time [4]. For patients with advanced ccRCC or relapse, many molecular-targeted drugs have been used as first-line therapies. Nevertheless, outcomes are poor due to the side effects of these agents and individual differences in individual drug sensitivities [5].

It is a fundamental challenge for cells to copy their genetic material for daughter cells accurately. Once this process goes wrong, genomic instability occurs [6]. The level of genomic instability is reflected in nucleotide instability, microsatellite instability, and chromosome instability [7]. DNA damage can be caused by mistakes in DNA replication caused by genotoxic compounds or ultraviolet and ionizing radiation. Incorrect DNA replication can lead to mutations or blocked replication, leading to chromosome breakage, rearrangement, and dislocation [8]. Genomic instability is an essential source of genetic diversity within tumors. Oncogene expression drives proliferation by interfering with regulatory pathways that control cell cycle progression. Genomic instability produces large-scale genetic aberrations but also increases point mutations in protein-coding genes. The estimated mutation rate in tumors is an order of magnitude higher than that of typical healthy tissue. Genomic instability also changes as tumors develop, and this trait could become a target for treatment [9].

Recent advances in sequencing technology have revealed that only 2% of the human genome codes for proteins [10]. Non-coding RNAs are classified into small non-coding RNAs and long non-coding RNAs according to their size. Long non-coding RNA (lncRNA) predominate. LncRNAs play central roles in many cellular mechanisms, including regulation of cell processes [11]. They also regulate pathophysiological processes through gene imprinting, histone modification, chromatin remodeling, and other mechanisms [12, 13]. LncRNAs also play essential roles in cancer. They are involved in chromatin remodeling and transcriptional and post-transcriptional regulation through various chromatin-based mechanisms and interactions with other RNA species [14, 15]. LncRNA imbalances can alter functions such as cell proliferation, anti-apoptosis, angiogenesis, metastasis, and tumor suppression [16]. Depending on their positions and distribution in the genome, lncRNAs directly or indirectly affect the transcription of various proteins through transcriptional and post-transcriptional changes, some of which may mediate tumor inhibition or promotion [17].

Because chemotherapy, radiation therapy, targeted therapeutic agents, and immune checkpoint inhibitors do not function well in many ccRCC patients, investigators need to develop new treatment options and further identify prognostic biomarkers and therapeutic targets ccRCC. LncRNA screening and model building based on gene instability in ccRCC may represent an important research strategy.

## Materials and methods

### Data collection

We downloaded clinical information, protein-coding RNA expression data, lncRNA expression data, and somatic mutation information for clear renal cell carcinomas from The Cancer Genome Atlas (TCGA) database (https://portal.gdc.cancer.gov/) [18]. We considered 507 ccRCC samples with paired lncRNA and mRNA expression profiles, survival information, and clinical information.

We divided all ccRCC samples into a training set and a test set. The training set included 254 samples for the creation of a clinical outcome lncRNA risk model. The test set included 253 patients, used to validate the predictive ability of the prognostic risk model. We provided detailed data on TCGA clear cell renal carcinoma (Supplementary Table 1). Meanwhile, we calculated the tumor mutation burden (TMB) in the samples and estimate the average number of mutations in the tumor genome [19].

### Mining lncRNAs related to genetic instability

First, we calculated the number of somatic mutations in each sample. The samples with the number of somatic mutations in the top 25% were defined as the genomic unstable (GU)-like group. The samples with the number of somatic mutations in the bottom 25% were defined as the genomically stable (GS)-like group. We combined the lncRNA expression matrix of TCGA-KIRC with the GU and GS groups and obtained each group’s lncRNA expression matrix. We then conducted a difference analysis on these two lncRNAs matrixes; |fold change| > 1 and false discovery rate adjusted *P* < 0.05 were defined as genome instability-associated lncRNAs. The result of genome instability-associated lncRNAs difference analysis is displayed in Table 1.

**Table 1:** lncRNAs related to genetic instability

lncRNA	logFC	pValue	fdr
ZNF582-AS1	−1.067	2.78E-10	1.32E-07
LINC01558	−1.926	3.72E-10	1.32E-07
GAS6-DT	−1.559	1.40E-09	3.30E-07
AL035661.1	−6.144	3.65E-07	5.17E-05
AC016405.3	1.376	6.21E-05	2.20E-03
AC005082.1	−2.200	7.72E-05	2.53E-03
LINC01187	−8.158	8.16E-05	2.53E-03
AL031123.1	−2.978	9.95E-05	2.82E-03
LINC02471	1.040	1.08E-04	2.94E-03
AC079466.1	3.017	1.30E-04	3.18E-03
LINC01606	−4.716	1.41E-04	3.33E-03
LINC01230	−7.363	1.74E-04	3.98E-03
AC148477.4	−4.784	2.06E-04	4.38E-03
LINC01896	−6.526	3.24E-04	6.20E-03
AC144831.1	−1.297	5.42E-04	8.53E-03
LINC00284	− 3.520	1.14E-03	1.32E-02
AL139351.1	1.105	1.40E-03	1.52E-02
LINC01234	2.170	1.63E-03	1.67E-02
LINC00460	1.276	1.75E-03	1.75E-02
MIR222HG	−1.371	2.25E-03	1.87E-02
AP000924.1	1.031	2.15E-03	1.87E-02
LINC00645	−2.101	2.24E-03	1.87E-02
OSTM1-AS1	1.425	3.90E-03	2.71E-02
AC130371.2	−1.271	4.05E-03	2.73E-02
INSYN1-AS1	−5.803	6.46E-03	3.94E-02
AC087636.1	1.708	8.52E-03	4.93E-02

*lncRNA* Long non-coding RNAs; *logFC* log_2_Fold Change; *fdr* False discovery rate

### Functional enrichment analysis and GSVA

We calculated the correlations between each protein-coding gene and the lncRNAs obtained as described above using the Pearson correlation coefficient method [20]. We ranked these protein coding factors in descending order according to the correlation and selected mRNAs with the top 10 correlation coefficients as the co-expression coding genes of lncRNA. Using functional analysis of these co-expressed coding genes, we analyzed the biological functions of these genetically unstable lncRNAs. Gene Ontology (GO) enrichment was performed using the clusterProfiler package in R, version 3.6.3 [21]. GSVA, which is estimated in an unsupervised manner, has a higher ability to detect changes in pathways in the sample population [22]**.** We downloaded the GSVA score from the molecular signatures database (http://software.broadinstitute.org/gsea/msigdb) to construct the gene set. Then, GSVA score was performed for each gene set in each sample using GSVA R software package.

### Statistical analysis

We used Euclidean distances and Ward’s linkage method to perform hierarchical cluster analyses [23]. We used univariate Cox proportional hazard regression analysis to calculate the associations between expression level of genome instability-associated lncRNAs and overall survival. We performed multivariate Cox proportional hazard regression analysis to evaluate the weighting coefficient in the risk signature. The genome instability-related lncRNA (GILncSig) for overall survival was as follows: Log[h(ti)/h0(ti)] = a1X1+ a2X2 + a3X3 + ⋯akXk, where h(ti) is the function hazard, and h0(ti) is the baseline hazard, X1, X2, X3, ⋯Xk are covariates, and a1, a2, and a3 are the corresponding multivariate Cox proportional hazard regression coefficients. A detailed introduction can be found in our previous articles [24]. We were using the same best cut-off point (the point is determined by the samples, with the maximum sensitivity and specificity in time-dependent receiver operating characteristic (ROC) curve). Hazard ratio (HR) and 95% confidence interval (CI) were calculated using Cox analysis. The Kyoto Encyclopedia of Genes and Genomes (KEGG) [25] pathway of genome instability-related lncRNAs were identified using gene set variation analysis [22]. All statistical analyses were performed using R-version 3.6.3.

## Results

### Differences in long non-coding RNAs

**Fig. 1:**
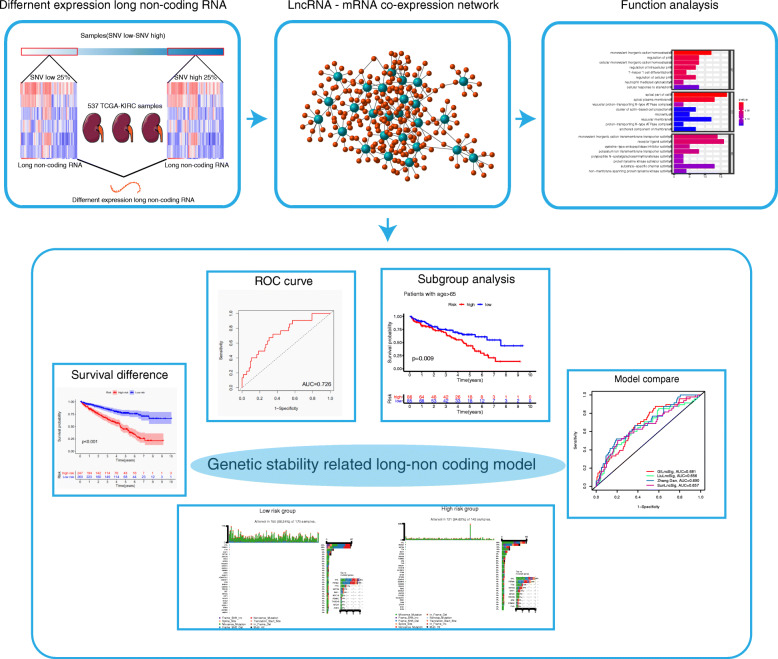
The design flow chart of this study. Clinical follow-up information of renal clear cell carcinoma, protein-coding RNA expression data, long non-coding RNA expression data, and somatic mutation information were downloaded from the TCGA database, and the samples were then divided into training sets and test sets. The samples were then divided into two groups for difference analysis according to gene mutation. According to the results of difference analysis, the overall samples were divided into gene stable group and gene unstable group by consensus cluster analysis. Then lncRNA-mRNA co-expression network was constructed, and the pathway analysis and GSVA scores were performed for this network. Then a COX regression prognostic model was established, and the model verification processes such as survival analysis, clinical subgroup analysis, tumor mutation burden analysis and model comparison were carried out

The design flow chart of this study was shown in Fig. 1.

**Fig. 2:**
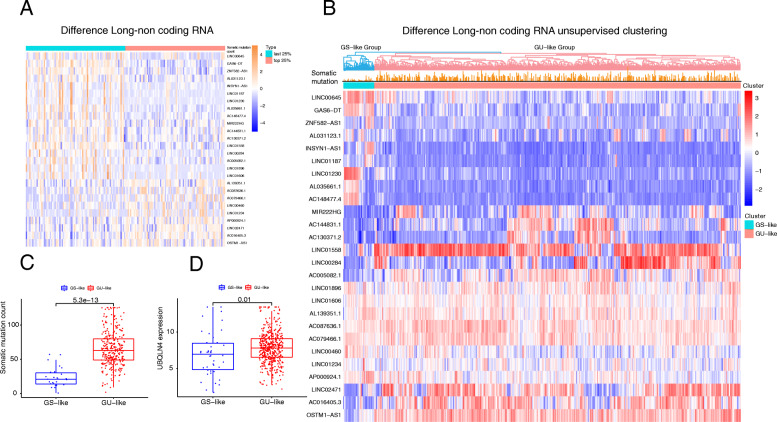
(**A**) Difference analysis of the group that Somatic cell mutations are in the top 25% between the group that Somatic cell mutations are in the last 25% in RCC. (**B**) Unsupervised clustering of GS-group and GU-group. (**C**) The difference of somatic cell mutation number between GS-group and GU-Group. (D) The different expression of UBQLN4 in GS-Group and GU-Group

To identify non-coding genes related to genome instability, we grouped them according to the number of somatic mutations. We placed the first 25% of somatic mutations (84 samples) into the genetically unstable group and then placed the final 25% of somatic mutations (84 samples) into the genetically stable group. We screened and obtained differential non-coding RNAs using the limma package. We screened a total of 26 non-coding differential RNAs, of which 17 were down-regulated, and nine were up-regulated (Table 1). The levels of differential non-coding RNA expression in both groups are shown in Fig. 2a.

### Genome instability-related lncRNA

We performed unsupervised clustering of all samples in KIRC based on the expression levels of these 26 lncRNAs (Fig. 2b). We obtained two clustering results, and the number of somatic mutations in the two groups was significantly different (Fig. 2c, P = 5.3e-13, Mann–Whitney U-test). Next, we compared the expression levels of the genomic instability driver ubiquilin4 (UBQLN4) in the GS-like and the GU-like groups (Fig. 2d) [26]. We found that the expression of UBQLN4 was significantly up-regulated in the genetically unstable group. We supplemented the correlation coefficient between UBQLN4 and other lncRNAs (Supplementary Table 2). Based on these results, we tested whether samples with different mutation levels could be distinguished based on expression levels of the 26 differential lncRNAs, and indirectly demonstrate that these lncRNAs may be related to genome stability.

### LncRNA-mRNA co-expression network

**Fig. 3:**
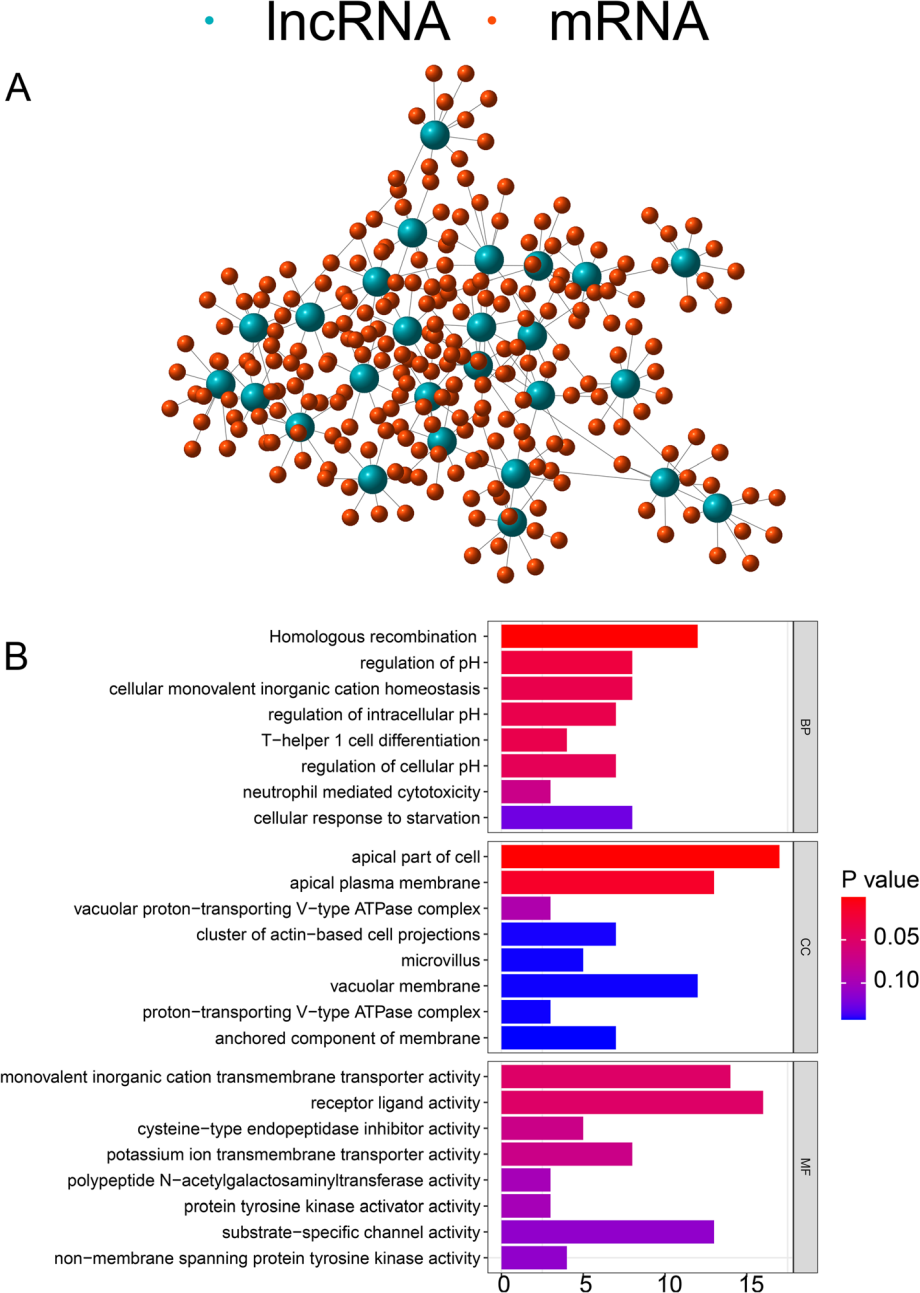
(**A**) The co-expression network of lncRNA-mRNA. Green stands for LncRNA and red for mRNA. The closer the relationship, the closer the connection. (**B**) Go analysis of the lncRNA-mRNA network. In the biological process, the network is mainly enriched in the monovalent inorganic homeostasis. In the cellular component, the network is mainly enriched in apical part of cell and apical plasma membrane. In the molecular function, the network is mainly enriched in monovalent inorganic cation transmembrane transporter activity and receptor ligand activity

Based on Pearson correlation coefficients, we determined the top 10 mRNAs that correlated with each lncRNA. We created a co-expression network lncRNAs and mRNAs (Fig. 3a). We then analyzed the function of the mRNAs in the co-expression module to determine the associated biological processes. GO enrichment demonstrated that these protein-coding genes are related to biological processes such as homologous recombination (Fig. 3b). This analysis suggests that the 26 genomically unstable non-coding RNAs may affect genome stability by regulating their co-expression networks. We found that these co-expressed protein-coding genes might regulate homologous recombination, thereby destroying cell stability. In total, we identified 26 non-coding RNAs related to genome instability.

### The genome instability-related lncRNA risk model

We clarified the lncRNAs and biological processes related to genetic stability. Next, we calculated the correlations between these lncRNAs and clinical survival phenotypes. We randomly divided 507 clear cell carcinoma samples with detailed follow-up information into training groups and validation groups. We constructed a multivariate Cox proportional hazard regression model for ccRCC in the training set based on 26 genomic stable state-related lncRNAs. The coefficients of the risk factors in the model are shown in Table 2. Risk model (GILncSig) = 0.095 * LINC00460 + 0.165 * LINC01234 + 0.152 * AL139351.1 + 0.177 * MIR222HG + 0.123 * AC087636.1–0.027 * LINC02471. We found that LINC00460, LINC01234, AL139351.1, MIR222HG, AC087636.1 were transparent risk factors. The higher their expression, the worse the overall survival of patients with renal cancer. LINC02471 is a protective factor for ccRCC. The higher its expression, the better the overall survival. We supplemented the univariate cox regression analysis coefficients of clinical features and risk scores, risk scores acted as independent prognosis factors(Supplementary Table 3). Meanwhile, we added the pearson-correlation coefficients of LINC01234 and tumor mutation burden in other types of cancers(Supplementary Figure 1). LncRNA expression patterns and the distribution of somatic mutation count distribution and UBQLN4 expression for patients in high- and low-risk groups are shown in Supplementary Figure 2.

**Table 2:** Multivariate Cox proportional hazard regression analysis results

ID	coef	HR	HR.95 L	HR.95H	***P***-value
LINC00460	0.095	1.099	1.010	1.196	0.028
LINC01234	0.165	1.180	0.984	1.414	0.044
AL139351.1	0.152	1.164	0.966	1.402	0.010
MIR222HG	0.177	1.194	1.002	1.422	0.047
AC087636.1	0.123	1.131	1.013	1.263	0.029
LINC02471	−0.027	0.973	0.934	1.014	0.048

*Coef* coefficient; *HR* hazard rate

### The verification and evaluation of lncRNA model performance

**Fig. 4:**
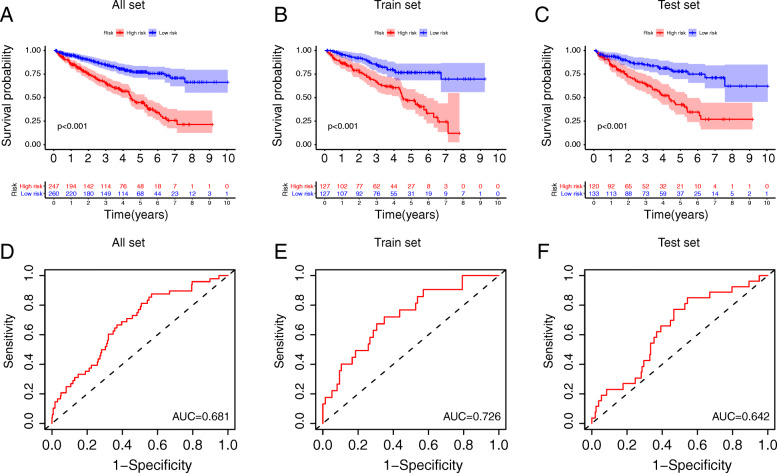
Survival analysis and ROC curve. (**A-C**) A COX prognostic regression model was established to calculate the scoring threshold, and a survival analysis was performed to assess the difference between the high-risk and low-risk groups. In the all set, train set and test set, patients in the low-risk group had a better prognosis than those in the high-risk group (*P* < 0.01). (**D-F**) The area under the ROC curve of the all set was 0.681, the area under the ROC curve of the train set was 0.726, and the area under the ROC curve of the test set was 0.642. The model shows good predictive ability

**Fig. 5:**
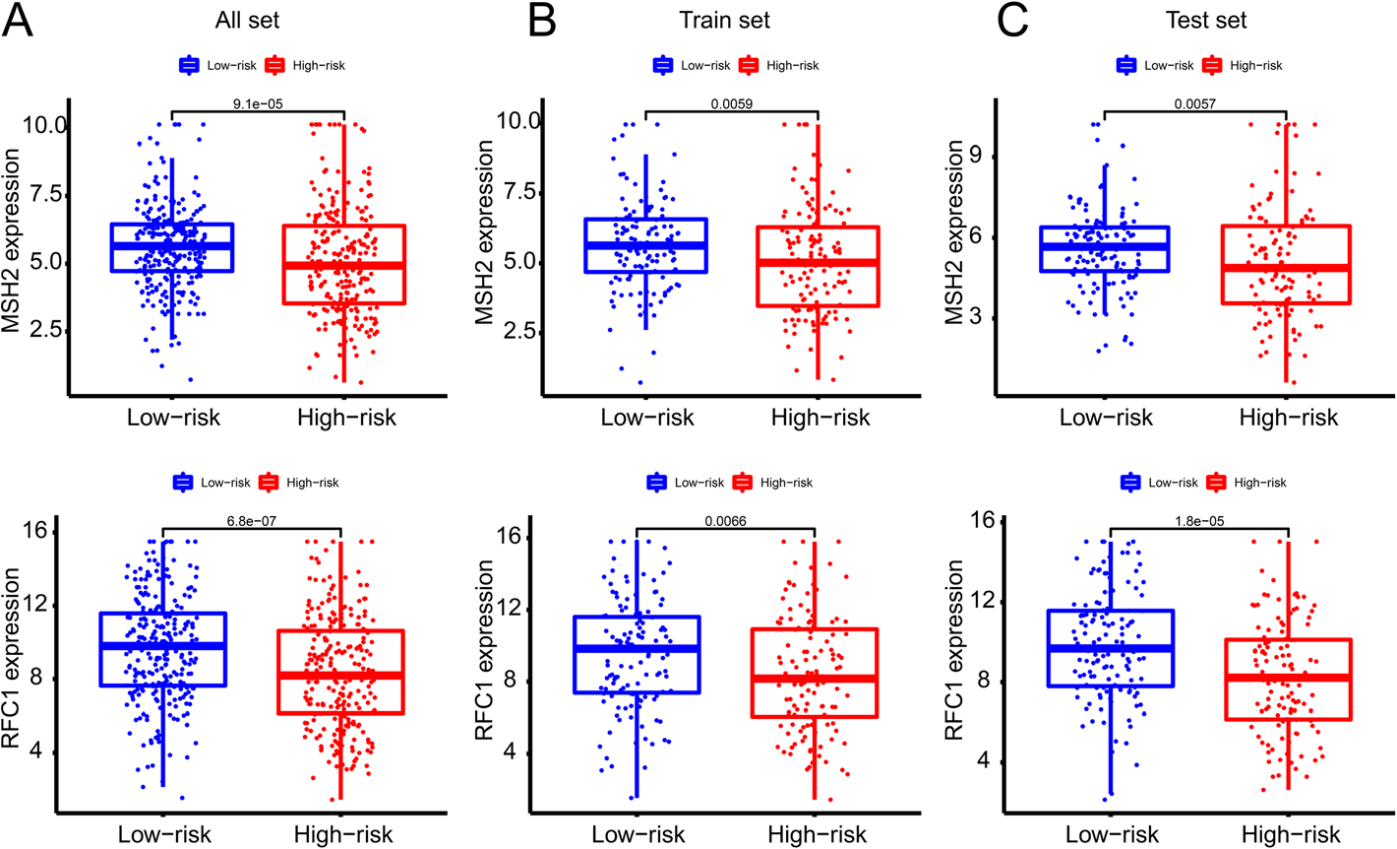
(**A-C**) The previously reported genetic instability related factor MSH2 showed significant differences in expression patterns between high-risk group and low-risk group in the all set (*P* = 9.1e-05), train set (*P* = 0.0059) and test set (*P* = 0.0057). (**D-F**) The previously reported genetic instability related factor RFC1 showed significant differences in expression patterns between high-risk group and low-risk group in the all set (*P* = 6.8e-07), train set (*P* = 0.0066) and test set (*P* = 1.8E-05)

Risk scores for each sample in the training and test sets were calculated using the GILncSig method. Patients were divided into groups according to the median risk score (0.853); patients in the higher risk group had a risk score > 0.853. We then calculated the survival difference between the high- and low-risk groups using survival analysis. In TCGA-KIRC cohort, we found that patients in the low-risk group had better clinical outcomes (Fig. 4a, P < 0.001). Patients in the low-risk group in the training set (Fig. 4b, P < 0.001) and validation set (Fig. 4c, P < 0.001) also had better survival outcomes. The area under the time-dependent ROC curve of TCGA-KIRC cohort was 0.681 (Fig. 4d). The area under the time-dependent ROC curve of the training set cohort was 0.726 (Fig. 4e). The area under the time-dependent ROC curve of the verification set cohort was 0.642 (Fig. 4f). MutS homolog 2 (MSH2) and replication factor C subunit 1 (RFC1) are involved in the process of mismatch recognition [27]. Comparison analysis showed significant differences in MSH2 and RFC1 expression patterns between the samples in the high- and low-risk groups (Fig. 5). Expression levels of MSH2 in the low-risk group were significantly higher than those of the high-risk group (*P* < 0.001, Mann–Whitney U-test; Fig. 3d). RFC1 also showed higher expression levels in low-risk patients than in high-risk patients (P < 0.001, Mann–Whitney U-test).

### Subgroups of the lncRNA model

**Fig. 6:**
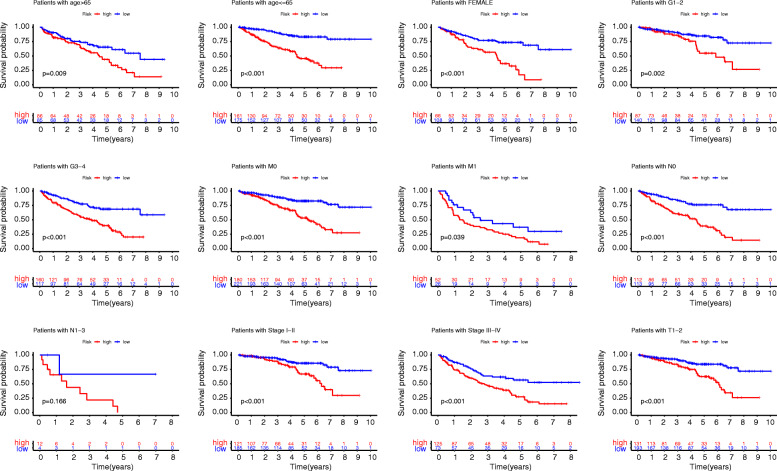
Subgroup analysis. The samples were divided into multiple clinical subgroups according to age, sex, stage, metastasis, and infiltration of lymph nodes. The results showed that in all clinical subgroups, the low-risk group had a better prognosis

We then obtained a stable genomic stability-related lncRNA prognosis model. To further analyze their performance levels in various subgroups, we conducted survival analysis. We found that subgroups of patients in the low-risk group achieve better outcomes (Fig. 6)(Supplementary Figure 3).

### Tumor mutation landscapes in high- and low-risk groups

**Fig. 7:**
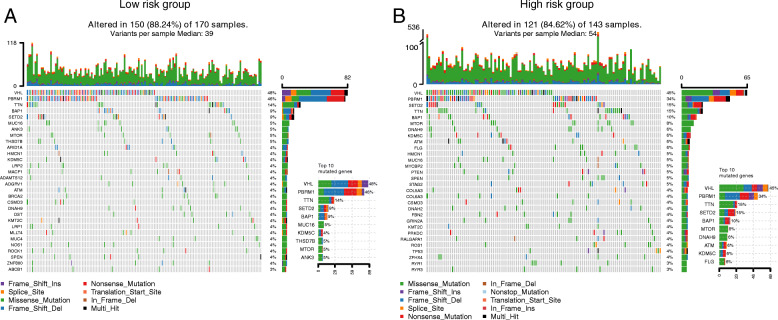
Waterfall map of gene mutation burden. (**A**) In the low-risk group, the mutation rate was 88.24%. The top three mutated genes were VHL, PBRM1 and TTN. (**B**) In the high-risk group, the mutation rate was 84.62%. The top five mutated genes were VHL, PBRM1, SETD2, TTN and BAP1

To compare mutations in the high- and low-risk groups, we drew a panorama of mutations in the two groups (Fig. 7). A total of 88.24% of the samples had mutations in the low-risk group. The top 10 mutated genes included VHL, PBRM1, TTN, SETD2, BAP1, and MUC16. The high-risk group’s mutation frequency (84.62%) was lower than that of the low-risk group (88.24%). The top 10 factors associated with mutations were the same as those of the low-risk group.

### Performance comparison in terms of AUC

**Fig. 8:**
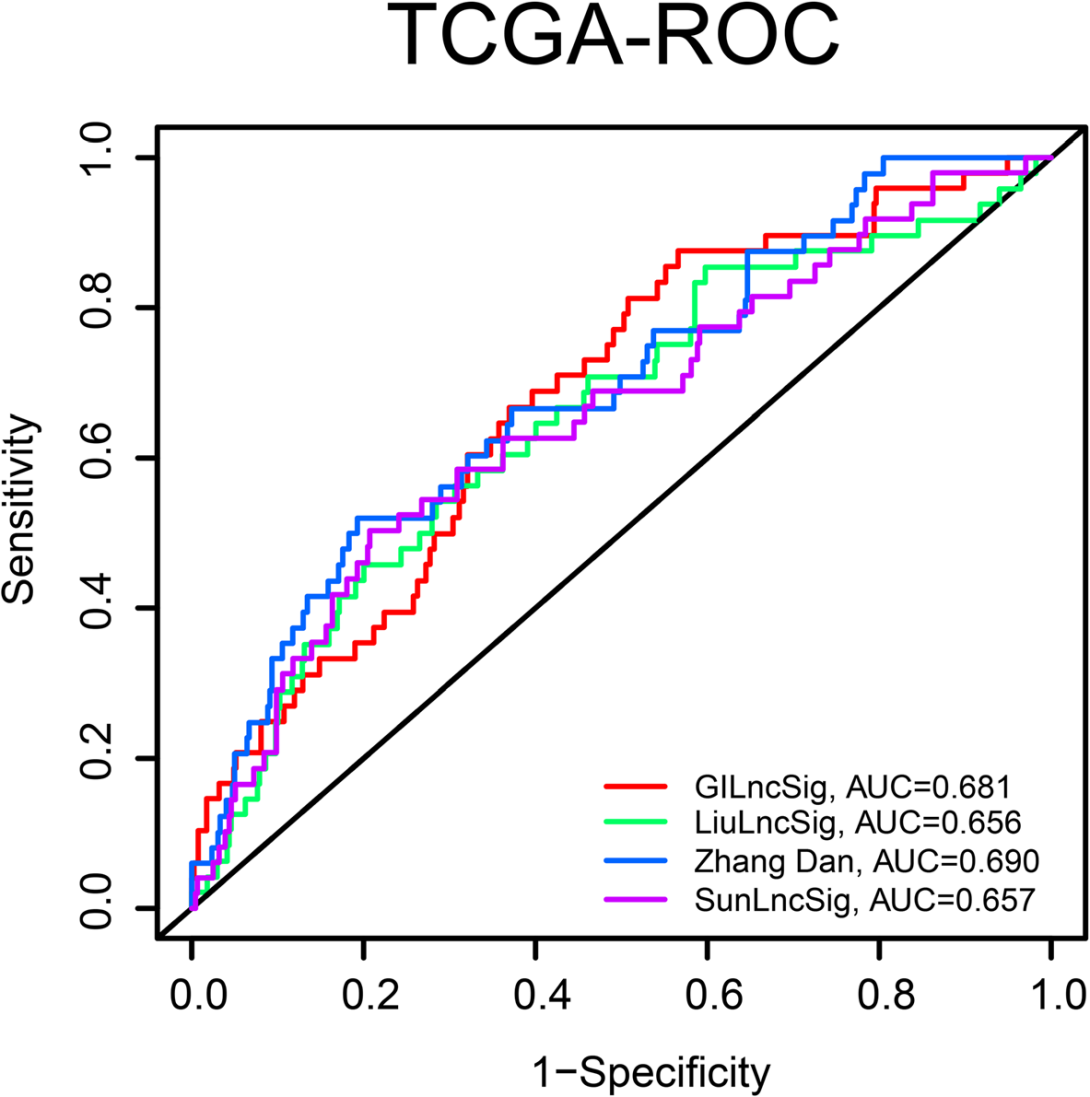
Model comparison. The model proposed in this paper is compared with the model of Liu et al., Sun et al., and Zhang et al., and the model presented in this paper has the highest ROC value, indicating the best evaluation ability

To determine the accuracy of clinical predictive models related to genome stability, we performed diagnostic test comparisons. Three recently published lncRNA signatures were involved in comparisons: the three-lncRNA signature derived from Zhang et al. (Zhang Dan) [28], the four-lncRNA signature derived from Liu et al. (LiulncSig) [29] and an immune signature derived from Sun et al. (SunlncSig) [30] using the same TCGA patient cohort. As shown in Fig. 8, the AUC of overall survival for the GILncSig was 0.681, which was significantly higher than those of SunlncSig (AUC = 0.657) and LiulncSig (AUC = 0.656) (Fig. 8). Although our model’s AUC was lower than Zhang Dan’s model, our training set score was 0.726.

### GSVA pathway correlation analysis

**Fig. 9:**
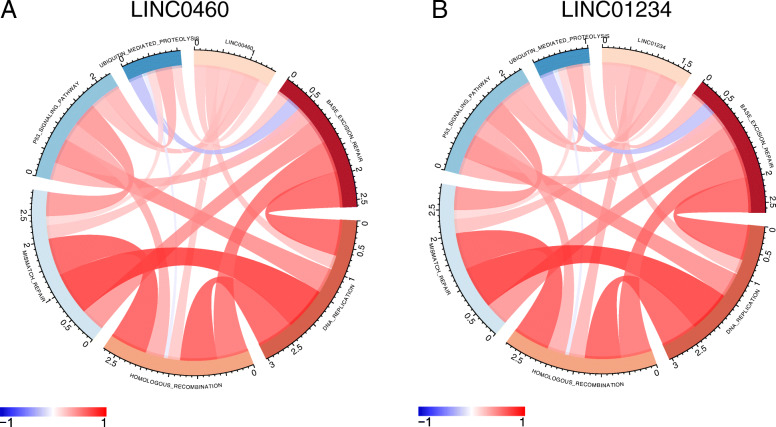
Correlation analysis of lncRNA and genomic instability related pathways. Red represents positive correlation and blue represents negative correlation. The selected pathways are: P53 signaling pathway, mismatch repair, homologous recombination, DNA replication and base excision repair

We obtained genome stability-related lncRNA in various somatic mutation groups; however, we believe that the lncRNA obtained based on differential analysis alone is insufficient to conclude that they are related to genome stability. Therefore, in this section, we obtained genomic stability-related pathway scores of each sample using the GSVA method. We calculated the Pearson correlation coefficients of these genomic stability pathway scores and the differences in lncRNA. We directly explained the pathways in which these factors regulate genomic stability. Figure 8 shows that the base excision repair pathway, the DNA replication pathway, homologous recombination, the mismatch repair pathway, the p53 signaling pathway, and ubiquitin-mediated proteolysis were related to LINC00460 and LINC01234. The interaction of these pathways appears to ensure the stability of the genome (Fig. 9). For these reasons, we believe LINC00460 and LINC01234 affect the stability of the genome by regulating these pathways. The correlation coefficient among genomic stability-related and lncRNAs were performed in Supplementary Table 4.

## Discussion

The genome structure’s relative stability is a prerequisite for the maintenance and continuation of the biological germline. It is crucial to ensure that a set of effective mechanisms is formed in the cell. There is a stable and accurate transmission of genetic information from generation to generation. Chromosome instability refers to the increased probability of acquiring chromosomal aberrations due to defects in processes such as DNA repair, replication, or chromosome segregation. Genome stability is closely related to the occurrence and progression of cancer [31–33]. Common DNA damage types include DNA base modification, DNA inter-strand, and intra-strand cross-links, and DNA single-strand and double-strand breaks [34]. Such DNA damage often leads to genome instability. Proteins related to DNA damage repair, DNA replication, and cell cycle checkpoints work together to ensure the integrity of the genome and the DNA structure’s integrity. However, mutations in these proteins can lead to the accumulation of mutations in chromosomes; as these mutations accumulate, they cause cancer and premature aging [32, 33, 35]. There is no accurate quantitative way to describe genome instability. Various efforts are underway to identify protein-coding genes and microRNAs related to genomic instability that predict outcomes [36–38].

Although we have made substantial efforts to identify lncRNAs related to genomic instability, whole-genome identification of lncRNA and its clinical research are still in their early stages.

Based on TCGA clear cell cancer cohort and the corresponding number of somatic mutations, we identified 26 differences related to the number of somatic mutations at the computational level. However, the analysis in computational biology is insufficient. Therefore, we combined clinical prognostic phenotype. A clinical predictive lncRNA model was constructed. We found that six lncRNAs in the model could be used as independent prognostic markers for renal cancer. According to our understanding, genome stability is closely related to levels of p53 mutations, DNA repair, and base mismatch repair. On account of the cumulative effect of these factors, normal cells gradually become cancer cells. According to our previous description, the six lncRNAs in the model should be closely related to these processes. Therefore, to verify this point of view, we performed GSVA gene set analysis and obtained the KEGG pathway scores corresponding to each sample. Then, the Pearson correlation coefficient test was performed using these pathways. LINC00460 and LINC01234 are the most relevant to these genomic stability pathways. We demonstrated that this method could screen candidate genome stability-related lncRNAs and identify the relevant pathways involved in these lncRNAs through GSVA analysis.

After a careful literature search, we found that the biological process of LINC00460 and LINC01234 in the GILncSig has not been reported to date. We found that the lncRNA LINC00460 was located on chromosome 13q33.2 and is a prognostic biomarker for esophageal squamous cell carcinoma [39] and renal carcinoma [28]. Another lncRNA, LINC01234, is located on chromosome 12q24.13. LINC01234 was found to regulate proliferation, migration, and invasion of ccRCC cells via the HIF-2α pathway [40]. Although studies have demonstrated the relationship between these two factors and outcomes of RCC, they do not explain the specifically related mechanisms. Finally, by analyzing the GSVA pathway, we found that they have the strongest correlation with the p53 pathway and affect the stability of the genome.

The transcription of lncRNA can affect the expression of neighboring genes [41]. Ephrin B2 (EFNB2), the neighboring gene of LINC00460, encoded the Ephrin family. Overexpression of EFNB2 is associated with malignant progression of tumors. It is expressed at high levels in head and neck squamous cell carcinoma and colorectal cancer [42], also promotes the growth of pancreatic ductal adenocarcinoma [43]. Knocking down EFNB2 can block tumorigenesis and establish tumor therapy [44].

RNA binding motif protein 19 (RBM19), the neighboring gene of LINC01234, Its function may be to participate in the regulation of ribosome biogenesis [45, 46]. Although there have been no specific studies linking RBM19 to cancer, other scientists have found that RBM19 is a gene expressed in the intestinal epithelium and is critical for intestinal morphogenesis [47].

There are some limitations to our study. First, we did not conduct cell or animal experiments. Second, we only identified 26 genomic stability-related lncRNAs; nevertheless, computational biology techniques demonstrated the connection between LINC00460 and LINC01234 and the genome stability pathway. Underlying regulatory mechanisms require further exploration.

In conclusion, we constructed a screening system for genome stability-related lncRNAs, and we identified 26 genomic stability-related lncRNAs, the detailed introduction of the 26 lnc RNAs was uploaded as Supplementary Table 5. We used these lncRNAs to predict outcomes in patients with ccRCC and found that these lncRNAs can be used as independent predictors. Finally, using GSVA pathway correlation analysis, we found that LNC00460 and LINC01234 are related to genome stability, and we indirectly demonstrated the appropriateness of this strategy.

## Supplementary Information

**Additional file 1.**

**Additional file 2.** (XLS 166 kb)

**Additional file 3.**

**Additional file 4.**

**Additional file 5.**

**Additional file 6.**

**Additional file 7.**

**Additional file 8.** LncRNAs related to genetic instability [48–70].

## Data Availability

“The datasets analysed during the current study are available in the TCGA repository, [https://portal.gdc.cancer.gov/]”.
