# Supplementary figures and images for: Genome instability-related long non-coding RNA in clear renal cell carcinoma determined using computational biology
Source: BMC Cancer. 2021 Jun 24;21:727. doi: 10.1186/s12885-021-08356-9 (PMC8229419; doi:10.1186/s12885-021-08356-9)

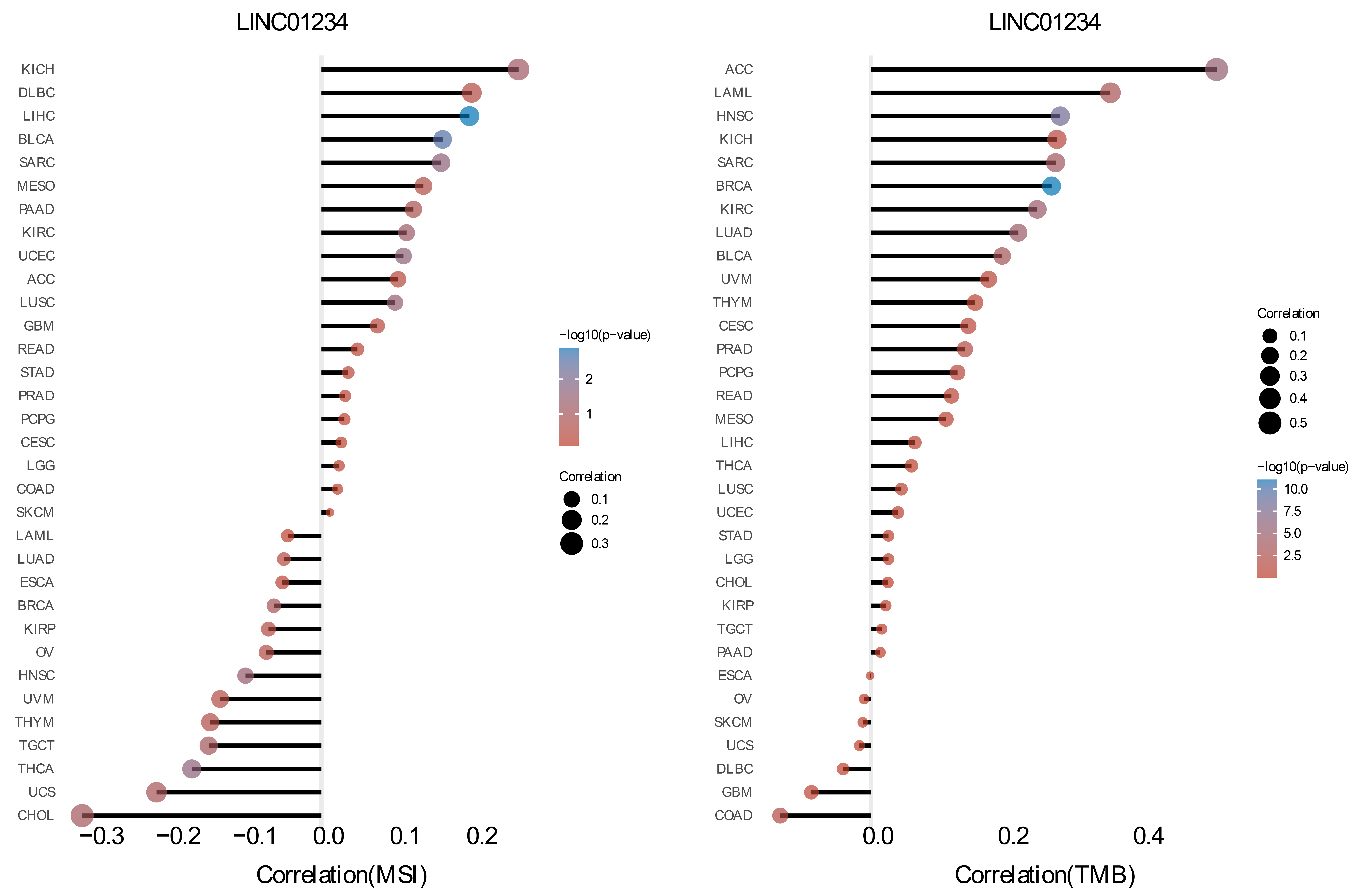

Supplement: Supplementary file 4 — Additional file 4. [file 12885_2021_8356_MOESM4_ESM.tif]

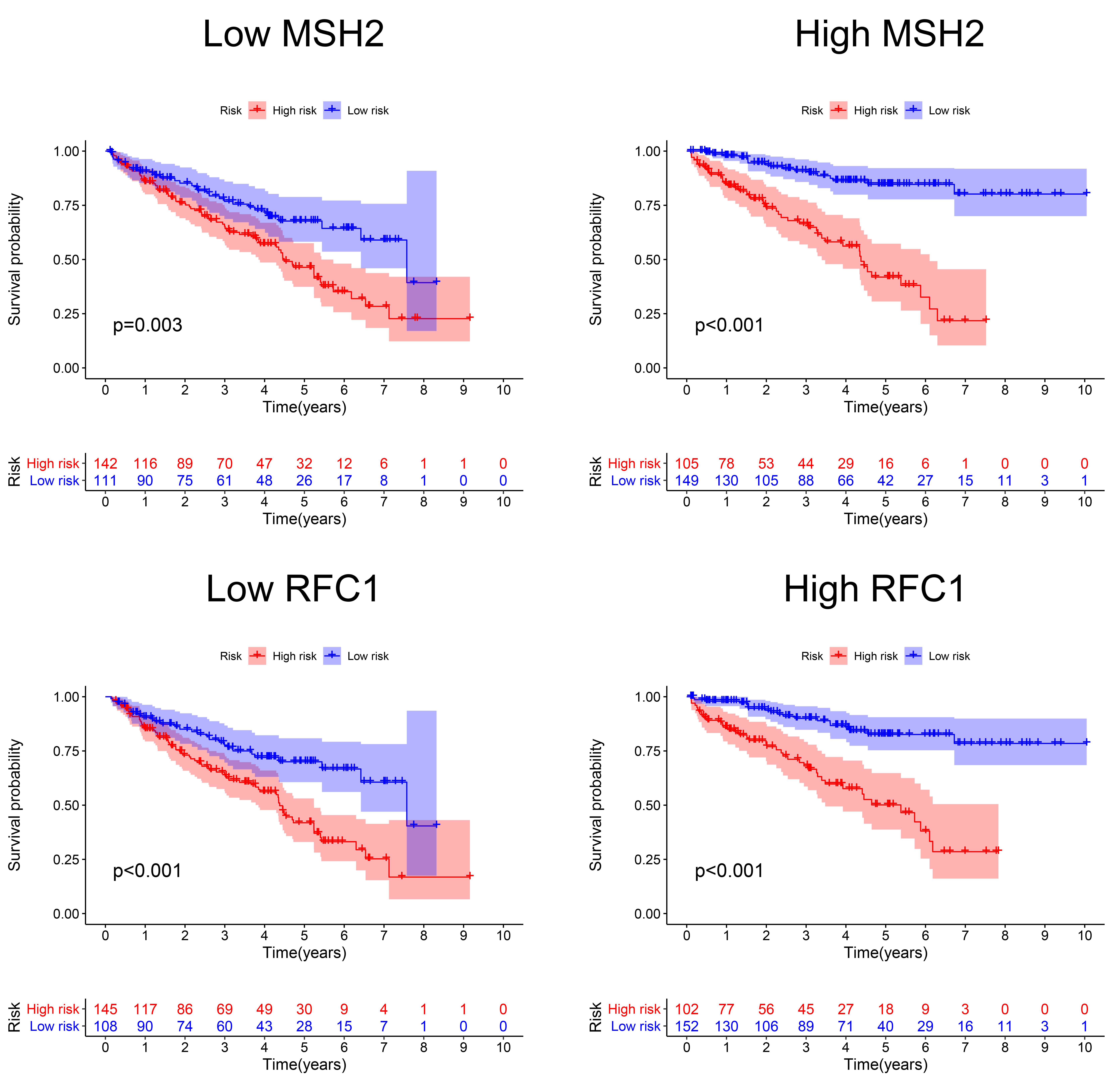

Supplement: Supplementary file 6 — Additional file 6. [file 12885_2021_8356_MOESM6_ESM.tif]
