# Supplementary material for: Genome instability-related long non-coding RNA in clear renal cell carcinoma determined using computational biology
Source: BMC Cancer. 2021 Jun 24;21:727. doi: 10.1186/s12885-021-08356-9 (PMC8229419; doi:10.1186/s12885-021-08356-9)
Supplement: Supplementary file 8 — Additional file 8. LncRNAs related to genetic instability [48–70]. [file 12885_2021_8356_MOESM8_ESM.docx]

| **Supplementary table 5. LncRNAs related to genetic instability** | |
| --- | --- |
| lncRNA | Function of lncRNA |
| ZNF582-AS1 | Acts as a tumor suppressor gene in Clear Cell Renal Cell Carcinoma and colorectal cancer[48, 49]. |
| **LINC01558** |  |
| GAS6-DT | Acts as a cancer-promoting gene in hepatocellular carcinoma[50]. |
| **AL035661.1** |  |
| AC016405.3 | Acts as an anticancer gene in glioblastoma[51]. |
| **AC005082.1** |  |
| LINC01187 | A genealogy-specific characteristic gene of chromogenic renal cell carcinoma[52]. |
| **AL031123.1** |  |
| LINC02471 | Acts as a cancer-promoting gene in papillary thyroid carcinoma[53]. |
| AC079466.1 | Acts as a cancer-promoting gene in hepatocellular carcinoma[54]. |
| LINC01606 | Acts as a cancer-promoting gene in gastric cancer[55]. |
| LINC01230 | Acts as  a novel modifier for PPARγ-mediated activation of Akt in endothelial function[56]. |
| **AC148477.4** |  |
| **LINC01896** |  |
| AC144831.1 | Plays an important role in severe asthma[57]. |
| LINC00284 | Acts as a potential biomark in papillary thyroid carcinoma, gastric cancer, triple negative breast cancer, ovarian cancer and oral squamous cell carcinoma[58-61]. |
| **AL139351.1** |  |
| LINC01234 | Acts as a potential biomark in hepatocellular carcinoma, renal clear cell carcinoma, non-small cell lung cancer, oral squamous cell carcinoma, esophageal cancer, gastric cancer, colorectal cancer, breast cancer[40, 62-66] |
| LINC00460 | Acts as a cancer-promoting gene in tongue squamous cell cancer, pancreatic cancer, cervical cancer, bladder cancer, cervical cancer, head and neck squamous cell cancer, etc [67]. |
| MIR222HG | Acts as a cancer-promoting gene in  glioblastoma and prostate cancer[68]. |
| AP000924.1 | Acts as a potential biomarker in triple negative breast cancer[69]. |
| LINC00645 | Acts as a cancer-promoting gene in  Glioma and [endometrial](G:/Dict/8.9.6.0/resultui/html/index.html#/javascript:;) [cancer](G:/Dict/8.9.6.0/resultui/html/index.html#/javascript:;)[70]. |
| **OSTM1-AS1** |  |
| **AC130371.2** |  |
| **INSYN1-AS1** |  |
| **AC087636.1** |  |
| lncRNA: Long non-coding RNAs; logFC: log_2_Fold Change; FDR: False discovery rate | |
